# Functional Magnetic Resonance Imaging (fMRI) and Music Therapy for Concussion: A Case Report

**DOI:** 10.7759/cureus.98823

**Published:** 2025-12-09

**Authors:** Teresa Hillier, Jaclyn Thiessen, Jim Chesnutt, Jeffrey M Pollock

**Affiliations:** 1 Diabetes and Endocrinology, Kaiser Permanente, Portland, USA; 2 Neuroradiology, Oregon Health & Science University, Portland, USA; 3 Family Medicine, Oregon Health & Science University, Portland, USA

**Keywords:** concussion, fmri, functional magnetic resonance imaging, mild traumatic brain injury, music therapy

## Abstract

Effective treatments for mild traumatic brain injury (mTBI) are limited and are insufficient to address patient symptoms and recovery. We report a case of an mTBI patient with debilitating visual symptoms who experienced profound symptom relief and increased functional capacity after music therapy (MT) (passive listening to specific music pieces). After other mTBI patients reported similar benefits, we conducted a functional magnetic resonance imaging study using visual paradigms to quantitatively measure the effects of this MT. MT reduced symptoms and increased visual cortex activation in the mTBI case. Our findings suggest that MT could have potential clinical effects for mTBI patients. More research is needed to establish the efficacy of this MT and identify the mechanisms by which this specific type of passive MT could be particularly therapeutic for mTBI patients.

## Introduction

Traumatic brain injury (TBI) causes physical, cognitive, and functional impairment in more than 2.5 million Americans [1]. Most TBI cases are defined as mild (mTBI), yet mTBI can have severe functional impacts and persistent sequelae [1,2]. Few known treatments improve mTBI symptoms: brief initial cognitive rest, graded sub-symptom threshold aerobic exercise, cervical/vestibular rehabilitation, and addressing headache and sleep sequelae [3,4]. Effective therapies are thus critically needed to provide symptom relief and facilitate better recovery for those who sustain mTBIs.

Music therapy (MT) is emerging as a promising treatment in acquired brain injury [5,6], particularly stroke [5]. MT is typically administered by certified professionals and can include the use of rhythm (e.g., for gait timing) and singing (e.g., for speech therapy) [5]. Passive listening to music is a form of MT that does not require a trained professional, is free and accessible to all, without adverse side effects, and has the potential to be tailored to specific clinical scenarios.

Here, we report a case of an mTBI patient who experienced symptom relief and subjective increased functional capacity after passive listening to specific musical pieces. In a functional magnetic resonance imaging (fMRI) study, we objectively measured the effects of this passive MT.

## Case presentation

Patient characteristics

An otherwise healthy 52-year-old female (T.H.) suffered her first mTBI after a motor vehicle accident. Symptoms included a new right occipital headache, poor binocular vision coordination, visual-spatial abnormalities, and executive processing fatigue exacerbated by visual tasks. Examination revealed impaired near-point convergence (18 cm), smooth pursuit, and saccades, and a right vertical hypertropia treated with prism reading glasses.

In the first week post-mTBI, despite cognitive rest, she had increasing headache, worsening visual symptoms, and hyperacusis: music she had previously found relaxing exacerbated symptoms. On Day 5, she discovered a genre of ambient meditation music with a relaxing, unstructured melody that provided immediate, temporary relief from her headache and allowed her to maintain focus on visual tasks while listening.

Later, she discovered two musical pieces [7,8] that had profound, distinct, and reproducible positive effects, which she likened to a "reboot." When she was experiencing debilitating visual symptoms, after listening to them once, these pieces immediately led to a deep, restful state. She would then slowly return to pre-injury levels of subjective functioning with lessened headache. Effects initially persisted for 1-2 hours; the duration of effects grew over time. MT immediately before bed also subjectively ameliorated severe mTBI-related sleep difficulties (including reducing sleep latency). Ideal listening conditions were: alone in a quiet place, eyes closed, lying down or sitting, no other activity or input, and listening through wired headphones or a stereo (noise-canceling headphones exacerbated symptoms).

fMRI procedure

In 2018, the patient participated in a single-session, four-minute visual fMRI protocol at baseline and after 5 and 25 minutes of passive MT to a 25-minute musical piece [7].

**Figure 1:**
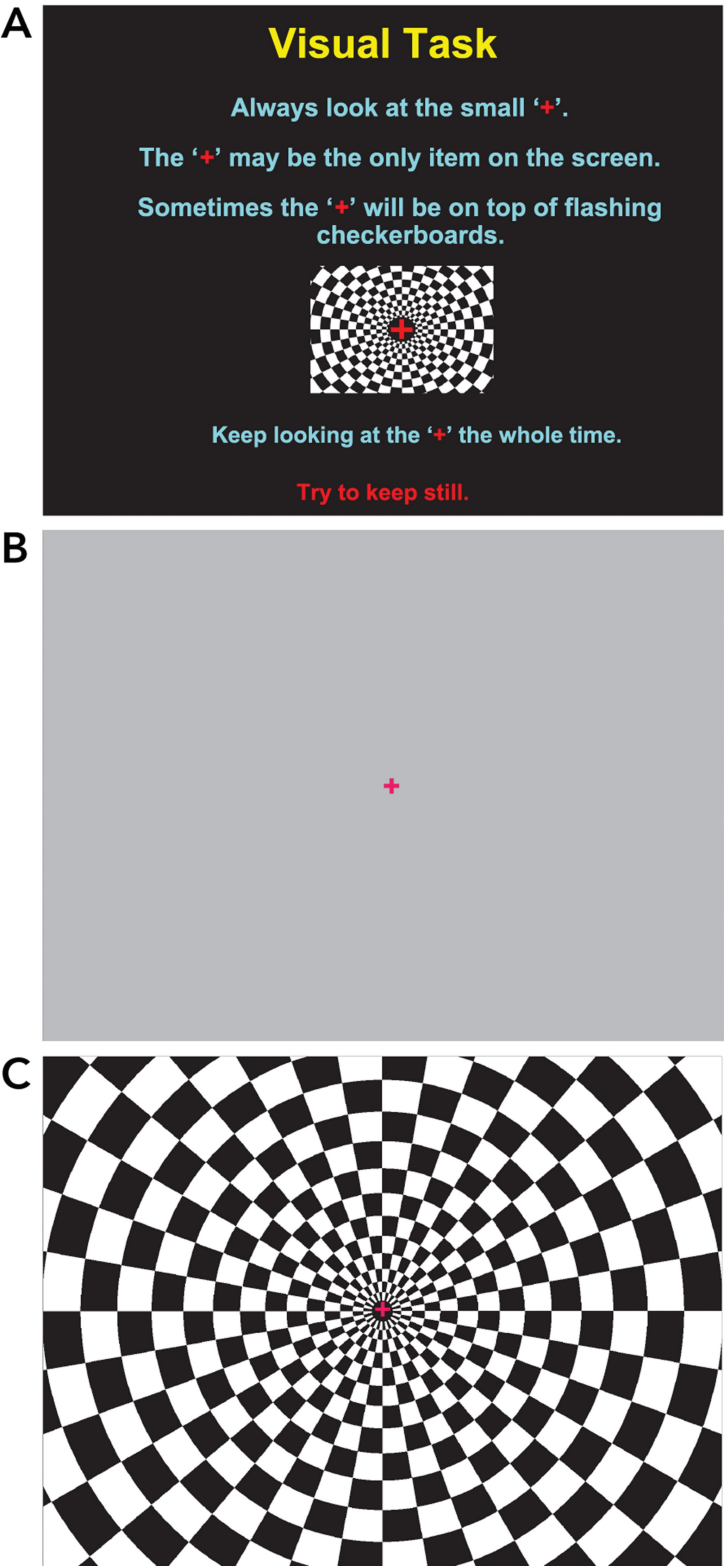
fMRI visual paradigm Visual images displayed on the monitor for the patient during fMRI visual paradigm testing (visual checkerboard paradigm): (a) Instruction slide, then alternating screens every 15 seconds of (b) reference slide and (c) visual activation slide for 4 minutes. fMRI: Functional magnetic resonance imaging.

The patient underwent fMRI on a 3 Tesla Philips MRI using the in vivo paradigm delivery system. An axial 3D T1 reference series was obtained, followed by the standard in vivo fMRI visual paradigm with a 26-cm field of view. She was instructed to focus on a central cross in front of a rotating checkerboard pattern alternating every 15 seconds with a resting non-stimulating background (Figure 1). 

**Figure 2:**
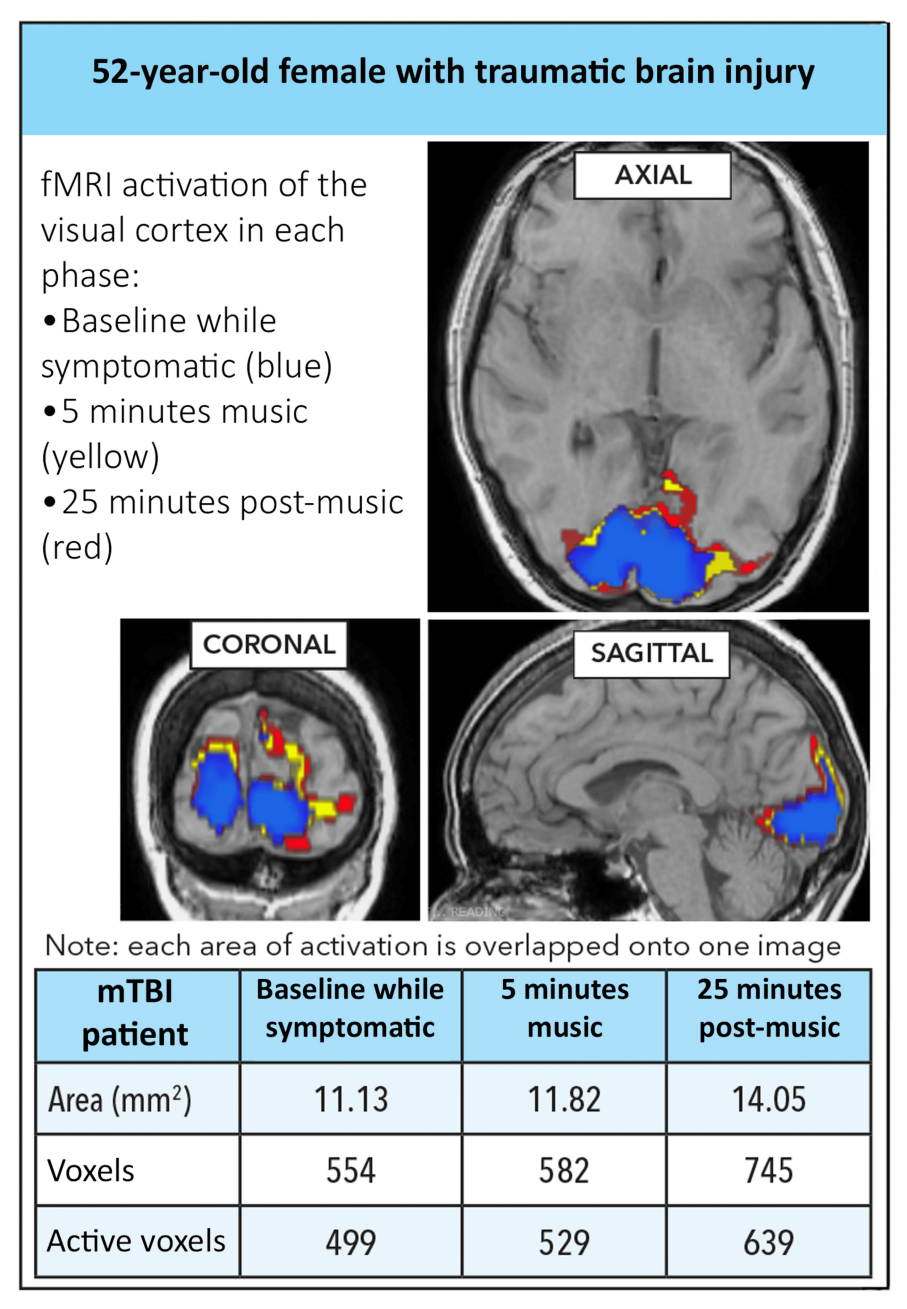
fMRI axial, coronal, and sagittal Images for the index TBI case at baseline (blue), after 5 minutes (yellow), and 25 minutes (red; completion) of passive listening to the MT. Corresponding visual cortex activation area, total voxels, and active voxels are presented in the table at the bottom of the figure, showing an increasing area of visual cortex activation over time with MT. fMRI: Functional magnetic resonance imaging; MT: Music therapy; TBI: Traumatic brain injury.

All scans were postprocessed identically using the DynaSuite software package to co-register, align, and process the acquisitions with a threshold p-value of 0.05. Occipital cortical regions of interest were automatically drawn on the axial images using the fMRI tool in the DynaSuite software, which automatically creates a region of interest surrounding the area of activation; activation area and total number of active voxels were recorded (Figure 2).

Imaging results

The patient was symptomatic at baseline and had difficulty maintaining fixation. She subjectively reported somewhat improved fixation and headache after 5 minutes of MT and marked improvement after 25 minutes. Activation area and voxels corresponded with subjective symptoms, demonstrating significantly increased activation at 25 minutes (28% increase in active voxels from baseline, 499 to 639, p<0.001). 

## Discussion

Increased occipital cortical activation and improved symptoms with MT in an mTBI case are consistent with previous evidence that MT can be an effective treatment for stroke and moderate-to-severe TBI; however, evidence of treatment efficacy in mTBI is limited [5,6,9]. These results, combined with reports by the index case and other patients, suggest that this type of MT may relieve mTBI symptoms and could potentially also promote long-term recovery from mTBI. The mechanisms of these effects are unknown, but it is plausible that MT could facilitate the brain’s recruitment of healthy neural pathways to replace or restore damaged ones. Such neuroplasticity after injury is complex, varies in time course from milliseconds to months, and has been observed in a myriad of neurologic diseases [10]. MT could affect these processes in one or several complementary ways. Intriguingly, the case’s cortical response to MT appeared to be greater on the asymptomatic left side with initial fMRI testing; similar contralateral recruitment has been observed after stroke [11]. One possible mechanism for long-term effects could be through the positive effects of MT on sleep reported by the case: most mTBI patients report new-onset sleep disruption [12], and sleep is paramount to mTBI recovery as the brain system clears metabolic waste primarily during sleep [4,11-13]. Moreover, it is plausible that MT promotes recruitment of auditory and other normal pathways to enhance recovery of the injured visual system.

While speculative and not yet demonstrated in mTBI populations, electroencephalography (EEG) evidence suggests that music can induce highly specific and often complex cortical phase synchronization [14,15]. Cortical activity (by EEG) is enhanced by low-frequency (bass) sounds; bass sounds boost selective neural locking to the beat [15]. The brain can also synchronize with a prolonged binaural beat (when slightly different frequencies are played in each ear, e.g., a 6-Hz difference increased theta rhythm by EEG) [16]. As a deficiency in timing tasks may be integral to mTBI sequelae [17], it is possible that music and other therapies that promote timing could harness the brain’s inherent plasticity to adapt neural networks [10,18]. 

In summary, MT could have widespread clinical effects for mTBI patients. While we await clinical research to systematically explore these effects and their mechanisms, providers and patients should be empowered to consider MT as a safe, low-cost, and easily accessible potential therapy option for patients struggling to find a path to recovery after mTBI. More research is needed to identify the mechanisms by which this specific type of passive MT could be particularly therapeutic for mTBI patients. 

## Conclusions

In this single-subject report, we demonstrate that passive MT delivered subjective functional and symptomatic recovery in a single patient with chronic mTBI. This clinical benefit was accompanied by measurable changes in brain activity, evidenced by increased fMRI activation, which provides potential neurobiological support for the intervention. These positive preliminary results should be validated in prospective, adequately powered future clinical studies.
